# Choice and Design of Adjuvants for Parenteral and Mucosal Vaccines

**DOI:** 10.3390/vaccines3010148

**Published:** 2015-03-05

**Authors:** Huub F. J. Savelkoul, Valerie A. Ferro, Marius M. Strioga, Virgil E. J. C. Schijns

**Affiliations:** 1 Cell Biology and Immunology, Wageningen University, Wageningen, P.O. Box 338, 6700 AH Wageningen, The Netherlands; E-Mail: schijns.virgil@gmail.com; 2 Strathclyde Institute of Pharmacy and Biomedical Sciences, University of Strathclyde, 161 Cathedral Street, Glasgow G4 0RE, UK; E-Mail: v.a.ferro@strath.ac.uk; 3 Department of Immunology, Center of Oncosurgery, National Cancer Institute, P. Baublio Str. 3b-321, LT-08406 Vilnius, Lithuania; E-Mail: strioga@gmail.com; 4 ERC-Belgium and ERC-The Netherlands, 5374 RE Schaijk, The Netherlands

**Keywords:** adjuvant, immunology, mechanisms

## Abstract

The existence of pathogens that escape recognition by specific vaccines, the need to improve existing vaccines and the increased availability of therapeutic (non-infectious disease) vaccines necessitate the rational development of novel vaccine concepts based on the induction of protective cell-mediated immune responses. For naive T-cell activation, several signals resulting from innate and adaptive interactions need to be integrated, and adjuvants may interfere with some or all of these signals. Adjuvants, for example, are used to promote the immunogenicity of antigens in vaccines, by inducing a pro-inflammatory environment that enables the recruitment and promotion of the infiltration of phagocytic cells, particularly antigen-presenting cells (APC), to the injection site. Adjuvants can enhance antigen presentation, induce cytokine expression, activate APC and modulate more downstream adaptive immune reactions (vaccine delivery systems, facilitating immune Signal 1). In addition, adjuvants can act as immunopotentiators (facilitating Signals 2 and 3) exhibiting immune stimulatory effects during antigen presentation by inducing the expression of co-stimulatory molecules on APC. Together, these signals determine the strength of activation of specific T-cells, thereby also influencing the quality of the downstream T helper cytokine profiles and the differentiation of antigen-specific T helper populations (Signal 3). New adjuvants should also target specific (innate) immune cells in order to facilitate proper activation of downstream adaptive immune responses and homing (Signal 4). It is desirable that these adjuvants should be able to exert such responses in the context of mucosal administered vaccines. This review focuses on the understanding of the potential working mechanisms of the most well-known classes of adjuvants to be used effectively in vaccines.

## 1. Introduction

Vaccines are widely considered the most important contribution of immunology to human and animal health. Vaccines used to prevent infectious diseases are generally based on a live-attenuated version of the pathogen, a non-replicating inactivated pathogen or a purified or recombinant subunit antigen. Live-attenuated vaccines and recombinant vectors are highly immunogenic, as they can infiltrate the host tissue, continue to replicate and are able to induce inflammation, similar to the pathogen itself. These activities, however, can result in the induction of side effects of these vaccines. These vaccines stimulate an excellent immune response that is nearly as good as that caused by an infection with the wild-type pathogen. Live microorganisms provide continual antigenic stimulation, giving sufficient time for memory cell production. In the case of viruses or intracellular microorganisms, where Th1 and CD8+ cytotoxic T cell-mediated immunity is usually desired, attenuated pathogens are capable of replicating within host cells. On the other hand, killed vaccines, in addition to purified subunit, peptide and DNA vaccines, lack these activities and are, thus, considered safer. However, these types of vaccines, in general, require additional factors to induce an effective immune response. Killed organisms and purified antigens commonly stimulate responses dominated by antibodies, otherwise known as a Th2 response. This antibody response may not generate optimal protection against some organisms. As a result, vaccines that contain killed organisms or purified antigens usually require the use of adjuvants to maximise their effectiveness. Adjuvants may however cause local inflammation, and multiple doses or high doses of antigen increase the risk of producing hypersensitivity reactions. In addition, the latter are often more sensitive to proteases and nucleases that destroy them before uptake by APC. Live-attenuated viral vaccines may be more potent than killed vaccines, and recombinant DNA technology can be used to make them safer [1,2]. For example, the introduction of attenuating mutations in the viral polymerase protein PB2 of the prevalent influenza strain and substitution of the wild-type gene could constitute a useful vaccine candidate that keeps pace with the antigenic shift of the virus [3,4]. To compensate for the low immunogenicity of non-replicating vaccine antigens, adjuvants are widely used and administered together with the antigen to induce and enhance antigen-specific immune responses in order to improve the clinical efficacy of the vaccine. Antigens together with an adjuvant form the vaccine, which in combination need to meet the approval of regulatory authorities. In order to obtain better safety profiles, non-replicating inactivated vaccines and subunit vaccines, as opposed to replicating live-attenuated virulent strains, are preferred in order to elicit protective immunity (especially in immunocompromised individuals), despite their limited immunogenicity [5,6].

Nowadays, in vaccinology, a wide variety of natural and/or synthetic molecules and targeted delivery systems are used as adjuvants to ensure the induction of the desired, strong immune responses, mostly in combination with weakly immunogenic inactivated vaccines or recombinant vaccine antigens. Some adjuvants may induce innate immune responses by up-regulating the expression of co-stimulatory molecules on dendritic cells (DC) and activate these APC by pathogen recognition receptor (PRR) engagement, resulting in enhanced local inflammation and subsequent effective triggering of adaptive immune responses [7]. Multiple administrations elicit immunological memory to provide long-term protection (months to life-long, depending on the vaccine) against future exposure to a particular virulent pathogen. As a result, the memory response is associated with a 10^3^- to 10^4^-fold increase in the frequency of antigen-specific B-cells, according to the clonal selection theory [8].

Currently, there are only a few adjuvants that are included in licensed human vaccines, including prominent examples, like: alum, MF59^®^ (oil emulsion), a squalene-based adjuvant system 03 (AS03^®^) and AS04^®^ (monophosphoryl lipid A, MPL + alum). The vaccine response usually recruits all components of the innate immune response, as well as the cellular (DC and T-cells) and humoral (vaccine-specific antibodies) components of the adaptive immune system to induce protective immunity. This state of immunity may wane with advancing age, due to immunosenescence, particularly in T-cells, and thus, repeated (booster) immunisations are often required to maintain full protection [9].

While vaccination to many antigenically stable pathogens is generally effective, there are still no viable vaccines for a number of important global pathogens, like human immunodeficiency virus-1 (HIV-1), several bacterial diarrheal pathogens, tuberculosis and malaria [10,11]. Therefore, novel approaches to vaccine development are required, which include improved and better targeted immune responses triggered by novel and rationally-designed adjuvants. Newly-developed adjuvants and rationally-designed vaccines need to incorporate recent insights in the innate immune response and the induction of a highly vaccine-specific adaptive immune response. We provide an overview of the working mechanisms of several parenteral and mucosal vaccine adjuvants and how these adjuvants can contribute to optimal and long-lasting protective immunity.

## 2. Immune Mechanisms of Adjuvant Action

The innate immune system consists of particular cells, like neutrophilic granulocytes, DC, macrophages (Mφ) and natural killer (NK) cells, in addition to soluble molecules, like cytokines, chemokines, acute phase proteins and the complement system. It is characterised by fast kinetics (minutes to hours), omnipresence in the entire body and by its use of germline-encoded receptors (PRR), including toll-like receptors (TLR) recognizing conserved molecular patterns on viral, bacterial and fungal infectious particles (pathogen-associated molecular patterns, PAMP) that are essential for microbial pathogenicity. This quick innate response limits the spread of an infection and, following antigen uptake and presentation, induces an antigen-specific immune response. On the other hand, the adaptive immune system consists of APC, like DC and Mφ and T- and B-lymphocytes, as well as humoral factors, like (different) cytokines and chemokines, and antigen-specific antibodies. It reacts slower (days), shows a profound degree of antigenic specificity down to the level of single amino acids (generally five to eight amino acids) on a protein (epitopes), develops long-term specific memory and, for antibodies, displays an amplification and increased functionality by affinity maturation (ten- to hundred-fold increases in K_D_ (M^−1^)) and heavy chain isotype switching (from IgM to IgG subclasses and IgA antibodies). This reactivity ensures the final clearance of an infection and prolonged protection, due to memory T- and B-cells, which represent the hallmark of vaccination [12,13].

By definition, adjuvants are considered molecules or formulations that enhance the vaccine-specific immune response without contributing directly to the immune protective effect. They function in many cases by modulating the activity of the innate immune response with ultimate consequences for the downstream adaptive immune response, resulting in antigen-specific protective immunity and long-lasting vaccine-specific immune memory formation. For the induction of an effective immune response to a viral vaccine, several signals are required: Signal 1 for the peptide derived from the vaccine, which is presented in the context of MHC surface antigens; Signal 2 for the induction of co-stimulatory (CD80 and CD86) interactions among APC interacting with peptide-specific T-cells; Signal 3 for immunomodulation of the quality of the ensuing effector response, mainly by the bidirectional delivery of cytokines (IL-1β, IL-6, IL-4, IL-10, IFN-γ); and Signal 0, reflecting activation of the innate immune response, which is necessary to induce increased expression of Signals 1 and 2 [14,15]. Vaccine-associated adjuvants contribute directly to the expression and regulation of all of these signals, but each adjuvant has an individual strategy. Adjuvants are therefore not always mutually exclusive. In addition, adjuvants may affect Signal 4, which instructs the homing receptors for elicited effector cells (CXCR4, CXCR5, α4β1 and α4β7). DC may provide Signal 4, leading to the imprinting of chemokine receptor expression and effector T-cell homing characteristics [16,17]. Several classes of well-known vaccine adjuvant types and their (expected) mode of action, receptors and downstream immune pathways are listed in Table 1.

Historically, vaccine adjuvants were mainly developed in an empirical manner, based on the assumption that antigen adsorption would prolong immune stimulation *in vivo* [18]. Therefore, adjuvant activity has been based on chemical stabilisation and improved delivery of antigens to APC, and their processing and presentation of the antigen to T-cells. Activated APC then secrete immunomodulatory cytokines, enhancing the ensuing immune response and, thereby, decreasing the required vaccine dosage [19].

### 2.1. Signal 0 Facilitation

The germline-encoded PRR of the innate immune system recognise evolutionarily-conserved PAMP as signatures of invading pathogens, also called Signal 0. Many different PRR types are expressed on APC, and exposure to their relevant ligands induces a cascade of innate immune cell responses; thereby influencing the subsequent vaccine-specific response. PRR include several families of receptors, like membrane-associated TLR, intracellular nucleotide-binding oligomerisation domain (NOD) proteins, NOD-like receptors (NLR), RIG-I-like receptors (RLR), retinoic acid-inducible gene 1-like helicases (RLH) and C-type lectin receptors (CLR). These PRR can each recognise a group of homologous molecules, called homotopes or PAMP. The currently known PAMP are evolutionarily highly conserved molecular structures that identify a particular group of microbes (bacteria, viruses, fungi and protozoa) and that can bind secreted receptors (e.g., pentraxins) found in blood and lymph associated with complement activation or opsonisation activity, intracellular (e.g., NOD) and membrane receptors (e.g., CLR, TLR) on APC associated with endocytosis or induction of NF-κB and mitogen-activated protein kinase (MAPK)-dependent signaling pathways [20]. Examples are lipopolysaccharide (LPS), peptidoglycan, flagellin or unmethylated CpG DNA, or viral ssRNA, or dsRNA. As a consequence of ligand binding, activation occurs of transcription factors, like NF-κB and insulin regulatory factor (IRF)-3. Subsequently, this activation induces the secretion of cytokines and chemokines that largely determine the priming, expansion and polarisation of the vaccine antigen-specific responses. Ligand binding to several NLR members (NLRP3 and NLRC4) induces the formation of an inflammasome that is involved in the production of pro-inflammatory cytokines, like IL-1β and IL-18. These inflammasomes determine the induction of an innate immune response triggered by the presence of the adjuvant alum, but the mechanism of this action remains unclear, especially since the demonstration of inflammasome activity requires primary activation by microbial PAMP, which may not be present in each vaccine [21,22,23,24].

**Table 1:** Classes of well-known vaccine adjuvant types, their (expected) mode of action, related receptors and downstream immune pathways. IFA, incomplete Freund’s adjuvant; MPL, monophosphoryl lipid; MP59®, squalene-containing adjuvant; AS04®, adjuvant system 04; QS (QS-21) substance extracted from the bark of *Quillaja saponaria*; ISCOM, highly immunogenic immune stimulating complex; LT, Enterotoxigenic *Escherichia coli* (ETEC) heat-labile toxin; CT, cholera toxin; MDP, muramyl dipeptide.

Adjuvant	Immune mechanism (presumed)	Immune SIGNAL	(Innate) ligands or receptor	Adaptive immune response type
Alum- and oil-based emulsions including IFA, Montanide®, MF59®	Ag depot effect MHC presentation	1	Unknown	DC, recruitment Th2, neutralizing Ab Th1, opsonizing Ab CTL, MF activation
MPL + alum (AS04®)	DC activation + migration	1		B memory, Ab
Liposomes	Depot effect + APC modulation	1	C-type lectin Card9 (unknown)	Th1, Th2, Th17
Saponins, ISCOM	Antigen delivery and T helper polarization	1 and 2	MyD88-dependent TLR-independent Unknown receptor	Th1, Th2, CTL, Ab
PRR agonist TLR-, NLR-, RLR, RLH agonists	Innate immune cell activation	0 leading to 2	PRR, including TLR, NLR, RLR, RLH agonists	Various, including Th1 pathways
MDP (example)	NRLP3 inflammasome activation	2	NOD2	Various, including Th1 pathway
ISCOMs, QS21	T helper polarization	3	Unknown, Mincle receptor	Various, including Th1
LT, CT, mucosal delivery	Homing to mucosal tissue	4	GM-1	Mucosal IgA and T cell activity

Many immunostimulatory adjuvants principally work by being recognised by unique (combinations of different) PRR or scavenger receptors [25]. Each PRR responds with different intracellular signalling transduction pathways leading to complex interactions, which determine the strength of the co-stimulation signal (immune Signal 2) and the final outcome of the ensuing adaptive response. Hence, Signal 2 facilitating adjuvants mostly contain microbial components, often called “stranger” (non-self) signals, which determine their ability to stimulate an innate immune response. Such pathogen-derived components include bacterial endotoxin (LPS), mycobacterial components present in complete Freund’s adjuvant, single-stranded ribonucleic acid (ssRNA) and bacterial deoxyribonucleic acid (DNA). The latter include synthetic CpG DNA. Alternatively, innate immune cell receptors may also recognise so-called “danger” (damaged-self) signals, which are released in tissue that has been damaged, not necessarily by microbial infection, but by tissue injury in general and normally not seen by immune cell receptors. Examples include heat shock proteins (HSP) [26] or uric acid [27,28], but also nuclear host DNA [29,30,31], which has been shown to mediate the adjuvant activity of alum [32]. Various activators of the innate immune response are thus attractive adjuvants that can be employed to selectively induce a preferred type of immune response.

Besides these cellular effects, complement activation by adjuvant also contributes to successful induction of vaccine-specific antibody formation. Activation of the complement and its subsequent binding to follicular dendritic cells (FDC), resulting in the activation of FDC and enhanced germinal centre (GC) formation, give rise to greatly increased antibody responses associated with the development of recirculating memory B-cells and bone marrow homing long-lived plasma cells [33]. The mannose-binding lectin pathway of complement activation follows the interaction with glycans present on adjuvant components. Certain TLR agonists (Signal 0) can trigger both Signal 1 and 2 and, thus, control the generation of T-cell receptor (TCR) ligands from the phagosome. This ensures the concomitant presentation of both TLR-ligated microbial components and the phagocytosed antigen by the activated APC. Such vaccine adjuvants, with combined delivery of antigen and TLR agonists, induce higher avidity interactions between peptide-loaded MHC and vaccine-specific T-cell receptors. Antigen presentation is enhanced by the combined presence within the same phagosome of antigen and the employed TLR agonist [34,35].

Furthermore, other receptors on APC can function in antigen targeting, such as CLRs and triggering receptors expressed on myeloid cells (TREMs). CLR, including the mannose receptor and DC-SIGN, specifically bind mannose and N-acetylglucosamine sugar moieties on pathogens. This enables the binding to a range of bacteria, viruses and fungi. Further research into these receptors is warranted to establish their applicability as vaccine adjuvant targets [36,37,38]. If these receptors trigger cellular activation pathways, they can be categorised as Signal 0; otherwise, they may act only as targeting receptors of APC, eventually facilitating Signal 1.

### 2.2. Signal 1 Facilitation

Many adjuvants can also selectively stimulate the uptake of vaccine antigens by APC, like Mφ and DC, and will protect the antigens from tissue-determined rapid degradation and elimination, thereby facilitating Signal 1. Widely-used adjuvants, such as alum, oil/water emulsions, lipid-based vesicles and micro-particles, are all considered depot-forming delivery systems that slowly and continuously release the antigen from the injection site, for a prolonged time relative to injection of the antigen only. Thereby, they somehow stimulate the infiltration of immune cells, like neutrophils granulocytes, DC, Mφ and, also, finally, even T-cells, in the case of booster immunisations. The subsequent release of cytokines and local mediators induces a pro-inflammatory innate immune response that results in tissue damage and ultimately leads to activation of a protective adaptive vaccine-specific immune response. There is thus a need for the development of Signal 1 adjuvants that can induce this protective immunity without inducing severe tissue damage. The presence of concomitant signals of vaccine antigen and the co-delivered PAMP as a result of tissue damage or the presence of danger signals induces the release of critical cytokines (e.g., TNF-α, IL-1β and IL-6) and chemokines (e.g., IL-8) for the recruitment of inflammatory cells and the induction of local inflammation. Heat shock proteins (HSP) are recognised as a danger Signal 0, which bind and help fold native and denatured polypeptides into molecular chaperones, e.g., nitric oxide synthase and guanylate cyclase, and, thereby, affect vascular relaxation [39].

Extracellular and membrane-bound HSP, especially Hsp70, are involved in binding antigens and presenting them to the immune system. Therefore, HSP act as an immunogen, as well as an adjuvant, thereby inducing and regulating the innate immune response against pathogens [40]. HSP has also been shown to induce the maturation of APC and result in specific triggering of the acquired immune response [41].

The induction of a local innate response, induced by adjuvants that act as antigen delivery systems, results in an increased uptake of the vaccine by potential APC. A subsequent rapid or delayed translocation of free or APC-bound vaccine antigen into the draining lymph node will occur, which can trigger an adaptive immune response following the presentation of antigen. A further level of complexity is added by the difference in MHC-dependent individual responsiveness to the vaccine antigen, but also to the adjuvant. Polymorphisms in MHC class I and class II genes and their master regulators, CIITA and RFX, determine the individually determined responsiveness to the vaccine [42,43]. The resulting magnitude of the protective immunity can therefore not always be readily predicted.

### 2.3. Signal 2 Facilitation

Under physiological conditions, activation of the adaptive immune system does not lead to tissue damage and is controlled and regulated by the induced expression of co-stimulatory molecules, resulting in the local release of cytokines. Molecules and formulations that facilitate immune Signal 2 represent a diverse group of adjuvants, consisting of a group of physically and chemically unrelated molecules, e.g., saponins, ISCOMs and MDP (see Table 1). However, these molecules can enhance the intrinsic immunogenicity of the vaccine antigen without affecting their delivery mode and localisation, which represents Signal 1. The final, net immunomodulatory capacity of distinct adjuvant molecules and formulations, however, is closely linked to the particular antigen composition, and their combined efficacy cannot be readily predicted when considering the adjuvant in the context of another vaccine antigen. As a result, each vaccine antigen requires a distinct and precisely chosen or designed adjuvant to elicit the desired type and strength of immune response.

Human peripheral blood contains two main types of DC: myeloid DCs (expressing the integrin CD11c) and plasmacytoid DC (expressing the IL-3R α chain (CD123)). Both subsets express MHC class II molecules. DC are generated in the bone marrow and subsequently are trafficked, based on their expression of α4β7, to the blood, the gut and airway mucosa. Recently, retinoic acid (RA) was found to increase the interaction between DC and antigen-specific CD8 T-cells in the gut mucosa, thereby exhibiting its effective adjuvant capacity. Vaccine carriers that direct the DC to specific mucosal tissues provide an attractive new generation of vaccines [44].

Bacterial toxins, including cholera toxin (CT) and the heat-labile enterotoxin of *Escherichia coli* (LT), are able to induce vaccine-specific Treg cells (in particular, the antigen-specific Tr1 type) in addition to the induction of Th2 and/or Th1 cells [45,46,47]. Due to their production of IL-10, these toxins modulate DC activation by downregulating the expression of the co-stimulatory molecule, CD40, thereby inhibiting the activity of the NF-κB transcription factor and dampening potent T-cell responses. On the contrary, CT-treated blood-derived monocytes in the presence of GM-CSF and IL-4 result in MΦ (CD14^high^CD1a^l^°^w^) rather than DC (CD14^l^°^w^CD1a^high^), expressing high levels of MHC-I and MHC-II and CD80 and CD86 co-stimulatory molecules. These cells also produce larger amounts of IL-1β, IL-6 and IL-10, but smaller amounts of TNF-α and IL-12 compared to DC in the absence of CT [48]. These findings suggest that CT results in enhanced expression of Signal 2 to developing T-cells and promotes their activation. In addition, cAMP-inducing bacterial A-B toxins also trigger IL-10 production and subsequent generation of Tr1 cells and, at the same time, inhibit CD40 expression and reduce NF-κB activity [49].

Certain TLR agonists, while acting as potent adjuvants, also induce IL-10-secreting Treg, while concomitantly stimulating the IL-12-dependent development of Th1 cells. This implies that the induction of a specific immune effector response simultaneously triggers a natural inhibitory response likely to dampen and regulate excessive effector cells. Blockade of this inhibitory response stimulates immune effector triggering. Therapeutic vaccination, resulting in CTLA-4 blockade, PD-1 blockade or depletion of Treg, has been shown to be effective in treating tumours. DC-based vaccines that induce suppression of IL-10-producing Treg by selective inhibitors of MAPK-p38, while simultaneously enhancing IL-12-producing Th1 cells, showed enhanced efficacy based on improving Signal 2 facilitation [50,51].

### 2.4. Signal 3 Facilitation

APC loaded with vaccine antigens will process and present peptides derived from the antigens within draining lymph nodes. These antigen fragments presented on the surface of APC can be recognised during a cognate interaction with specific CD4+ helper Th cells. As a result, both the APC and the T-cells will be activated and mutually secrete cytokines (IL-12 and IFN-γ, respectively). The local cytokine pattern, in addition to the expression of co-stimulatory molecules and local chemokines attracting other cytokine-producing cells, collectively will determine the differential outgrowth of subsets of distinct antigen-specific Th cells, including Th1, Th2, Th17, Th9, Th22 and, also, regulatory T-cell (Treg) populations, representing Signal 3 [52]. Immunomodulatory adjuvants, including alum and chitosan, are not only delivery systems affecting immune Signal 1, but they also modulate DC activity and enhance vaccine-specific Th2 responses and their associated cytokines, IL-4, IL-5 and IL-13, which profoundly stimulate B-cells to produce specific antibodies and, thereby, influence the quality of the adaptive immune response (Signal 3). These adjuvants have been extensively tested and found to be safe for human use. Furthermore, (biodegradable) micro- and nano-particles and particulate forms of antigen in general have strong effects on humoral and cellular vaccine-specific immunity by modulating the activity and antigen-presenting capacity of APC [53,54].

The addition of particular cytokines to the vaccine preparation containing adjuvant can selectively steer the resulting cellular and humoral immune response in a desired Th cell and antibody direction and can boost the efficacy of poorly immunogenic vaccines in humans and domestic animals [55,56,57,58,59]. Cytokines, however, have highly specific activity and are active in the pM range, and therefore, the dosing is critical to avoid the induction of immunosuppression. The immune system uses cytokines to fine-tune immune responses in a tightly regulated manner and cannot deal easily with bolus cytokine doses. Selected cytokines have an enhancing activity on antibody production (e.g., IL-4, IL-5, IL-6), while others induce heavy chain isotype class switching of vaccine antigen-specific antibodies (IL-4, IFN-γ, IL-10, TGF-β, IL-13). Such IgG antibodies can diffuse or be actively transported into particular sites (e.g., lamina propria, secondary lymphoid organs, skin), bind selectively to Fc receptors on monocytes (IgG1) or act as powerful activators of the complement cascade (IgM). IgA antibodies are present on mucosal surfaces by active epithelial transcytosis employing the polyIg receptor. Hence, cytokines might be beneficial when protective immunity is largely based on adequate antibody production, e.g., pertussis and tetanus vaccines. Since cytokines show strongly pleiotropic activity (having differing effects on different target cells) and since they work in complex networks with inherent additive, synergistic and antagonistic effects, combinations, rather than individual cytokines, will be effective *in vivo*, while the addition of single cytokines may not be the most practical approach.

Tregs can inhibit the development or effector phase of protective immune responses triggered by vaccines, which makes these cells viable targets to enhance the immunogenicity of vaccines. Vaccines targeting the interaction between CCR4 expressed on Tregs and its ligands, CCL22 and CCL17, can transiently inhibit the recruitment of Tregs at the site of vaccination, providing a sustainable target for rational adjuvant design [60,61].

Widely-used adjuvants, like incomplete Freund’s adjuvant, squalene-based oil-in-water emulsions, CpG oligodeoxynucleotides (ODN) and alum, are considered potent. Their mechanism of action, however, remains largely unknown. This can be based on TLR, or NLR, or the combination of agonistic activity (ODN), enhanced DC uptake (squalene), the production of cytokines, like type I interferons (IFN-α,β), IFN-γ, IL-2 and IL-12 (IFA, CpG), and inflammasome induction (alum). Collectively, such adjuvants regulate both innate and adaptive vaccine-specific immune responses [62]. The problem of the short half-life (minutes) of recombinant cytokines has been overcome by extending the half-life by technical advances, like encapsulation into liposomes and employing cytokine expression vectors that can enable co-delivery with DNA vaccines. In experimental systems, cytokines, like IFN-α, IFN-γ, IL-2, IL-12, IL-15, IL-18, IL-21, GM-CSF and Flt-3 ligand, potentiate vaccine-specific immune responses, but their applicability as potential vaccine adjuvants remains to be elucidated [63,64,65,66]. Despite proof-of-principle studies showing that cytokines can act as adjuvants in tumour vaccines, steep costs prevent their widespread use. A possible exception is GM-CSF, which has shown anti-tumour effects and improved patient outcome when applied in combination with suitable anti-tumour vaccines [67].

Cytokines, like IL-2, IL-6, IL-12, IL-15, Flt3 ligand, GM-CSF, MIP 1α and type I IFN, have all been used in nasal immunisation routes to induce potent protective immune responses, omitting the use of toxic CT and LT adjuvants when using subunit vaccines. Their application, however, is still limited to cell-mediated immunity. Nevertheless, CTB delivered intra-nasally was able to induceIL-6-dependent mucosal Th17 formation with associated IgA production, even under Th1- or Th2-promoting conditions [68]. Furthermore, in an influenza mouse model, there is data to support the use of cytokines as an adjuvant to induce mucosal antibody responses [69,70].

### 2.5. Signal 4 Facilitation

Interestingly, a poorly-chosen delivery adjuvant, like incomplete Freund’s adjuvant (IFA), may direct tumour antigen-specific effector immune cells to the vaccine injection site rather than the tumour, while the same tumour vaccine antigen injection in combination with a non-repository adjuvant has been shown to direct effector responses to the tumour [71]. The imprinting of homing signals of vaccine-induced immune effector cells is called Signal 4. Certain vaccine adjuvants, alone or administered via a specific route, may selectively influence the imprinting of Signal 4. The correct imprinting of Signal 4 is especially important for vaccines that require immune effector cells to travel to the anatomical locale where the effector immune response is needed, including tumours and chronic infections. For example, for mucosal tumours, mucosal imprinting seems important for correct homing of vaccine-induced CD8-positive T lymphocytes to inhibit the growth of mucosal tumours [72].

Protective vaccines are designed to mimic the kinetics of pathogen infection, with rapid initial virus replication and subsequent elimination to low levels. The timing of vaccine and adjuvant delivery remains crucial to induce effective DC cross-presentation and, thereby, determines the resulting immune response, as was shown by the enhanced CD8+ T-cell response, using co-administered α-galactosylceramide. Alpha-galactosylceramide (α-GalCer), an agonist of iNKT cells, induces the release of immunostimulatory cytokines, like IFN-γ, and the upregulation of costimulatory molecules. This contributes to the maturation of APC, the release of IL-12 by DC and the activation of NK cells, γδT cells, B-cells and CD4+ and CD8+ T-cells. These activities make a-GalCer a potent stimulator of innate immunity and a vaccine adjuvant, particularly in anti-tumour activities [73].

Vaccine antigens and adjuvant need to be co-localised in the same phagosome for efficient MHC class II presentation by APC [74]. Nevertheless, it has been shown that delivery of antigen and adjuvant separately in nanoparticles enhances anti-viral antibody titres and the ensuing germinal centre reaction [75]. Even repeated, non-adjuvant exposure to vaccine proteins can induce a state of immunological memory. Few studies have addressed the kinetics of the induced vaccine-specific T-cell immune response and T-cell-based effective immunity. Maximal tumour-specific T-cell immunity develops only late during or even after completion of the vaccine schedule. These kinetics could be based on boosting of pre-existing anti-tumour immunity. Measuring specific immunity after completion of vaccination could thus identify strong vaccine responders [76].

However, the use of vaccines and adjuvants has been extensively studied using non-mucosal immunisation routes. How these immunisation schemes and use of adjuvants can be optimised as effective mucosal vaccines remains to be further elucidated further [19]. The initial site of vaccine and adjuvant exposure also influences memory formation in cell-mediated immunity. In addition, DC from different sites influence the patterns of chemokine receptor and integrin expression and the tissue-homing specificities of primed CD8+ T lymphocytes (Signal 4). The CCL19- and CCL21-based migratory capacity of vaccine-activated DC towards effector sites for the protection of the place of the tumour can be fine-tuned by selected combinations of cytokines (like IFN-α and TNF-α) and TLR agonists, like flagellin, CpG DNA, lipoteichoic acid (LTA) and LPS [77].

## 3. Selected Adjuvants and Immunostimulation

Effective vaccination is historically based on the induction of binding antibodies or strain-restricted antibodies that neutralise the pathogen of interest and are protective. Nevertheless, the existence of pathogens that escape effective vaccine targeting and the required improvement of existing vaccines necessitate the development of novel vaccines based on the induction of protective cell-mediated immune responses. Elimination or masking of immunodominant epitopes of antigens induces the immune system to recognise previously subdominant epitopes, which may result in more broadly protective vaccine candidates.

In addition, newly-developed adjuvants should be optimised for the induction of cell-mediated responses, inducing protective immunity by enhancing vaccine-specific T-cell responses. This can be achieved by targeting specific innate immune cells, which facilitate proper downstream adaptive T- and B-cell immune responses, increasing the efficacy of the vaccine.

Adjuvants often behave like PAMPs that are recognised by innate immune system PRR. The main target of many adjuvants are DC, either by uptake through phagocytosis, forming a depot of these adjuvants and altering the cytokine production potential of these cells, leading to inflammasome formation (e.g., alum, incomplete Freund’s adjuvants, MF59, liposomes). Alternatively, adjuvants, like LPS and CpG-ODN, are known to enhance adaptive immunity via direct DC activation and maturation by enhancing the expression of MHC class II and co-stimulatory molecules (CD80/86) on APC, thereby indirectly stimulating vaccine-specific T-cell proliferation. For many existing or experimental adjuvants, more and more immunological mechanisms have been studied in recent years, using different immunological assays in animals or clinical studies. The use of distinct types of antigen in these investigations, the differences in administration regimens and dosing, make it difficult to make real comparisons between the studies. Nevertheless, each study adds value, leading to a better understanding of the mechanism of action of distinct adjuvant classes.

### 3.1. Oil-Based Emulsions

The mechanism of the adjuvant activity of water-in-oil (W/O) emulsions remains largely unknown [78,79], although pharmaceutical parameters, such as the type of oil, the droplet size, the type of surfactant and the oil-to-water ratio, are important parameters. Oil-in-water emulsions are phagocytosed by DCs and, thus, may not necessarily provide a depot-mediated adjuvant effect, but stimulate the innate immune response. Squalene is a cholesterol precursor, which is added to some adjuvant emulsions, like MF59 and AS03. Safety concerns associated with their local reactions and persistent oil residues drive the demand for alternatives. MF59, an oil-in-water emulsion containing a biodegradable squalene compound, was shown to increase the resulting antibody response to influenza vaccine due to enhanced vaccine uptake, trafficking to draining lymph nodes, recruitment of granulocytes and monocytes/Mφ and antigen presentation [80]. Non-toxic monophosphoryl lipid A (MPLA), derived from LPS, is a TLR4-targeting adjuvant component in AS04, which is approved for human use in licensed hepatitis B vaccine and human papillomavirus vaccine, Gardasil^®^ [81]. AS04 results in increased activation of the NF-κB pathway, stimulating memory T- and B-cells. This APC activity is dependent on the co-localisation of vaccine antigen and adjuvant in the draining lymph nodes [82]. In veterinary vaccines, many different oil-based adjuvants exist [74,75]. Oil-based emulsions are also used in licensed and experimental cancer vaccines [79,80].

### 3.2. Alum-Based Adjuvants

Alum refers to aluminium hydroxide or aluminium phosphate, to which the vaccine antigen can be absorbed and subsequently phagocytosed by APC to stimulate, in particular, humoral immunity and strong Th2 responses. The adjuvanticity of alum is considered to be based on three main features: depot formation, which ensures slow release of the antigen over a long time period; the induction of inflammation with subsequent recruitment of APC; and its ability to conform soluble antigen into particulate form, facilitating DC uptake of alum complexed with the vaccine antigen, which is considered to form a depot at the injection site, leading to the gradual release of antigen. This enhances the antigen presentation by APC, although this antigen-binding activity is not always necessary to potentiate the specific immune response [83]. The DC-dependent T-cell immunostimulatory properties of alum have been described as being partially dependent on inflammasome activation. Nalp3 is an intracellular recognition receptor (NLR), detecting damage-associated intracellular molecules (e.g., ATP or crystals of monosodium urate) and inducing the release of the pro-inflammatory cytokines, IL-1β and IL-18 [53,84]. The requirement of Nalp3 inflammasome activation for the adjuvant activity of alum is still debated. Some studies have shown the dependence of inflammasome activation upon priming of the APC with LPS, while other studies have shown caspase-1-driven release of IL-1β and IL-18 from DC [85]. In addition, alum may not use classical PRR to promote DC maturation. The dependence of alum activity on Nalp3 activation of alum was challenged by showing that Nalp3-deficient mice mount normal immune responses and by the observation that uric acid crystals alone, but not alum, augmented CTL responses to the injected antigen [86]. Alum-based adjuvants have a long history of use and a long safety record. However, alum does not induce proper CTL priming and is therefore not used as a cellular immunity vaccine adjuvant. There is no absolute support for the proposed Nalp3 activation pathway, the uric acid concept or the IL-1β or IL-18 dependence for alum adjuvant activity.

Aluminium adjuvant-containing vaccines induce inflammation, with inflammatory cells appearing at the injection site 2 h to 6 h after vaccination. The infiltrate is dominated by large numbers of neutrophils, macrophages and MHC-II-positive dendritic cells. These kinetics coincide with the increase in chemokines at the injection site. Macrophages are the main phagocytosing cells, while DC are the major APC of the vaccine antigen [87].

### 3.3. Damage-Associated Molecular Patterns

According to the “danger theory” of Matzinger, the immune system has evolved to focus primarily on danger, or tissue damage, rather than on microbial non-self-signals [88]. The theory divides antigens into two groups in the context in which these antigens are seen by the immune system: those associated with danger and those associated with harmless antigens. Innate immune cells recognise danger-associated molecular patterns (DAMPs) and PAMPs through interaction with pattern receptors (PRRs) and induce inflammatory responses. There are four classes of PRRs: toll-like receptors (TLRs), Nod-like receptors (NLRs), RIG-I-like receptors (RLRs) and C-type lectin receptors (CLRs). These PRR sense pathogen-derived factors and transduce activating signals into cells, triggering adaptive immunity against pathogens. Therefore, the ligands for PRRs, such as PAMPs and DAMPs, exhibit potent adjuvant properties that elicit adaptive immunity. Therefore, PRRs are considered to be receptors for adjuvants [89]. Stressed or damaged cells and tissues (hidden self) release danger signals, resulting in necrotic (but not apoptotic) death. As a consequence, danger signals lead to increased expression of co-stimulatory molecules on APC. Danger signals comprise mitochondrial and nuclear fractions of necrotic cells, as well as HSP, cellular DNA or uric acid, collectively referred to as danger-associated molecular patterns (DAMPs). Cytokines, such as type I IFN (IFN-α,β) produced by infected cells, are also DAMPs. Adjuvants, such as aluminium hydroxide, saponins and oil-based emulsions, induce tissue damage and the generation of DAMPs at the injection site and are therefore called Signal 2 facilitators [90,91]. For example, Marichal and co-workers [32] recently showed that alum causes cell death at the injection site, resulting in local DNA release, which is recognised by undefined DNA receptors, resulting in adjuvant activity.

### 3.4. Innate Immune Cell Receptor Agonists

Currently-used vaccines, using conventional adjuvants, induce a strong Th2 response with concomitant antibody formation. The current challenge, however, is to develop Th1-promoting adjuvants, resulting in subsequent vaccine-specific cellular immune responses against hepatitis, influenza, HIV and malaria. Vaccine preparations that incorporate pathogen-derived PAMP, as natural or synthetic agonists for PRR, could thus induce pro-inflammatory cytokines and chemokines in addition to type I interferons, collectively resulting in the removal of the specific pathogen by the host [92].

PRR pathways that induce strong cell-mediated immunity are attractive novel vaccine adjuvants for vaccines. Viral replication is associated with dsRNA formation that induces IL-12 and type I interferons characteristic of an innate immune response. This response can be mimicked by synthetic poly-(I:C), which interacts with TLR3 and RIG-1/MDA-5. Subsequent cross-presentation to MHC class II on APC improves CD4+ T-cells, which may help to generate specific cytotoxic CD8+ T-cells. The development of MPLA acting as a TLR4 ligand can induce a strong Th1 response. Flagellin, a TLR5 ligand, induces mixed Th1 and Th2 responses, and it is sometime fused to a recombinant vaccine antigen. The TLR7/8 pathway in DC can be activated by synthetic compounds, like imidazoquinolines (*i.e.*, imiquimod, gardiquimod and R848). This activation promotes Th1 responses by the production of IFN-α and IL-12 [93]. Specific CpG motifs containing ODNs, like CpG ODNs, such as ODN 1826 and ODN 2006, are recognised by TLR9, resulting in enhanced antibody production and a polarised Th1 response [94]. Muramyl dipeptide (MDP) present on bacterial cell walls triggers the activation of NOD2 and the NLRP3 inflammasome [95]. Certain combinations of different adjuvants can induce a stronger or more potent skewed immune response. Recent examples include combinations of TLR9 agonists or combinations of CpG ODNs with MDP or MPLA [7,96,97]. AS04 induces TLR4-mediated maturation of DC, resulting in enhanced migration to the draining lymph nodes and subsequent activation of vaccine-specific T-cells. CpG motif adjuvants induce enhanced expression of CD40, CD54, CD80, CD86 and MHC class II molecules and, therefore, enhanced antigen processing and presentation in plasmacytoid DC (pDC) [98,99].

### 3.5. Conjugated and Multivalent Vaccines to Improve Immunogenicity

Inherently weak immunogenic antigens, as included in inactivated and subunit vaccines, can be potentiated by chemical or molecular fusion of the vaccine antigen to highly immunogenic proteins (conjugated vaccine) or by mixing multiple antigens to create a multivalent vaccine. T-cell-independent antibody responses to capsular polysaccharides do not readily occur in children under the age of two years. For example, chemical coupling of bacterial polysaccharides to protein carriers provides carrier-derived peptides that induce T-cell-dependent anti-polysaccharide antibodies, facilitating bacterial opsonisation and constituting an effective vaccine [100].

Conjugating the capsular type b polysaccharide of *Haemophilus influenza* type b (Hib) to the tetanus toxoid as a protein carrier increased the immunogenicity of the vaccine. These conjugate vaccine-activated CD4+ Th cells enable isotype class switching of antigen-specific B-cells from IgM to IgG and induce antigen-specific memory B-cells [101].

Subunit polysaccharide or protein vaccines induce humoral, but most often not cell-mediated, responses, and thus, efficacious vaccines require immunodominant B-cell epitopes, as well as T-cell epitopes. By incorporating antigens into protein micelles, lipid vesicles (e.g. liposomes) or immunostimulating complexes (ISCOMs), a multivalent vaccine can be formulated that delivers many copies of the antigen into APC. This delivery route will ensure intracellular deposition of the vaccine antigen, allowing the protein to be processed by the endogenous pathway and antigen-derived peptides to be presented in relevant MHC class I molecules interacting with specific CD8+ Tc cells, eventually stimulating a vaccine-specific CTL response [4].

## 4. Mucosal Vaccines

The mucosae of the respiratory and gastro-intestinal system, with, on average, 80-m^2^ and 350-m^2^ surface areas, respectively, are the major entry ports for pathogens, and ideally, vaccines should be able to generate local protection against these infections. Most licensed vaccines, however, are mostly ineffective on immune cells in the mucosae, as they are primarily administered parenterally, and only a few new mucosal vaccines have been developed. Mucosal vaccines are simpler to administer, have less risk of transmitting infections and are potentially easier to manufacture. Oral vaccines exist against cholera, typhoid, polio and rotavirus and a nasal vaccine against influenza. Oral live vaccines, however, often have reduced immunogenicity in developing countries, because of malnutrition, aberrant intestinal microflora, concomitant infections and pre-existing immunity, as well as of host genetic factors that influence the immunogenicity of these vaccines. Recent developments in better delivery of vaccines on mucosal surfaces enable uptake by local APC, thereby generating protective mucosal immune responses [102].

Typically, for the gut immune system, there is an intense interaction between host cells, commensal and pathogenic bacteria, all of which can also have an impact on systemic immune responses. The local presence of defined DC subsets, together with the vitamin A metabolite, RA, and the presence of Tregs are crucial in regulating gut tolerance and homeostasis. RA, in conjunction with CD103+ DC and epithelium-derived cytokines, like APRIL and IL-10 or TGF-β, induces Treg generation, while at the same time inhibiting the expansion of Th17 cells, via the CXCR3+ mucosal DC subset. Gut microbiota generate signals that direct intestinal responses with effector T-cells against pathogens or, in the case of commensals, induce a state of tolerance via modulation of Tregs and the release of immunosuppressive cytokines, like IL-10 and TGF-β.

DC subsets are crucial in generating vaccine-specific immune responses. Mucosal DC are present in gut-associated tissues, including Peyer’s patches (PP), mesenteric lymph nodes (MLN) and the lamina propria (LP) of the villus mucosa. Some of these DC subpopulations can selectively induce the differentiation of Tregs. Orally-administered vaccine is mainly taken up by DC in the LP. Normally, intestinal DC are quiescent, and this state is linked to mucosal tolerance. However, these intestinal DC are responsive to inflammatory stimuli and, thus, able to present the vaccine antigen and induce T-cell priming, homing and protective immunity. When administered orally, highly immunogenic immune stimulating complexes (ISCOMs) containing Quil A preferentially target mucosal DC and, thus, may prove useful adjuvants for incorporation into mucosal vaccines [103,104].

For efficient vaccine-specific immune responses, it is crucial that the native pathogen or target antigen protein structure in the vaccine be retained, and therefore, proteolysis of the antigen before uptake by APC should be prevented. Most vaccine preparations are therefore injected, rather than administered by the oral route, exposing the vaccine to digestion in the gastro-intestinal tract. However, delivery systems, such as the lipid-based bilosome (vesicles containing bile salts), have been designed to protect the antigen from the extremes of pH [105]. The anatomy and the regulatory networks of the mucosal immune response pose unique levels of complexity for suitable adjuvants in mucosal vaccines [106]. Furthermore, for mucosal adjuvants, toxicity is an important safety issue. For parenteral use, thus far, only alum and some emulsions are clinically approved [1].

### 4.1. Mucosal Immunity *versus* Tolerance

A typical feature of the mucosal immune system is its capacity to distinguish between inducing an immune effector response when needed, while at the same time developing a state of oral tolerance to harmless (commensal and dietary) antigens [107]. Mucosal administration of vaccine antigens may induce T- and B-cell tolerance, rather than immunity, particularly without the use of an adjuvant. This holds true despite the fact that split tolerance exists, where T-cell immune responses co-exists with B-cell tolerance. Classical tolerance is dependent on the dose and the timing of antigen delivery, with “low-zone” tolerance referring to low antigen doses over prolonged periods of time and “high-zone” tolerance dealing with high doses of antigen overwhelming the immune system [88]. In both cases, antigens are specifically recognised and induce central or peripheral deletion of reactive T- and B-cells, while at the same time generating antigen-specific Tregs (called Tr1 cells). The slow release of antigen with a low dose and also rapid delivery of a high antigen dose at a mucosal surface are thus more likely to induce tolerance and, thereby, lose the benefit of using adjuvants. Frequently applied low doses of antigen or a single high antigen dose induce mucosal tolerance, while a single low dose or frequent high doses can break mucosal tolerance [108]. The addition of adjuvants, like CT, can break tolerance independent of the doses of the vaccine antigen, since CT is able to upregulate MHC class II and CD86 expression and IL-1β production, thereby skewing towards a Th2 response and IgG/IgA antibody production [48]. Thus, optimal antigen release kinetics must be controlled, particularly when designing mucosal vaccines while using suitable adjuvants.

### 4.2. Mucosal Adjuvants

The most commonly-used mucosal adjuvants are comprised of toxin-based adjuvants (LT, CT), immunostimulatory adjuvants (e.g. MPL, CpG, QS21) and particulate adjuvants (e.g., emulsions and ISCOM). LT and CT are potent, but also toxic, mucosal adjuvants. By using site-directed mutagenesis, toxins with reduced enzymatic activity were produced and used as adjuvants using oral, nasal and ano-genital immunisation routes. Intranasal vaccines for HSV, *Bordetella pertussis* and *Streptococcus pneumoniae* using such LT mutant adjuvants resulted in protection. The enhanced antigen permeation across epithelial barriers resulted in increased antigen uptake and presentation by APC, resulting in potent antigen-specific CD8+ Tc responses. Currently, oral vaccines are being optimised by adjuvant-mediated targeting M-cells in the PP of the intestine [109,110].

Polymeric vaccine carriers based on PLGA and chitosan are used for their capacity to adhere to mucosal surfaces, opening of tight junctions (and thereby facilitating antigen uptake) and their release kinetics of the vaccine antigen. However, their triggering capacity of innate immune responses has not been evaluated routinely. By using chitosan polymer nanoparticles during immunisation, stimulation of NALT mucosal secretory IgA, IgG, TNF-α, IL-6 and IFN-γ production could be achieved [111,112]. These polymers presumably work by stimulation of TLR2- or TLR5-mediated innate responses, thus making them attractive carriers of novel mucosal vaccines [113]. Furthermore, bacterial flagellin, being a ligand for TLR5 and NLR, can be employed as an effective mucosal vaccine [110].

In order to be efficient, mucosal vaccines must also provide systemic protection, thereby necessitating the use of adjuvants. CT and mutant *Escherichia coli* LT, which have reduced toxicity based on their inability to activate adenylate cyclase, showed improved performance as mucosal vaccines [114]. CpG-ODN act as stimulatory agents that might act in synergy with alum and CT as mucosal adjuvants. Pro-inflammatory cytokines, like IL-1α, IL-6, IL-12, IL-15 and IL-18, can act as mucosal adjuvants that induce mucosal CD8+ CTL and vaccine-specific IgA antibody production. Furthermore, chemokines, like MCP-1, increase mucosal IgA secretion and CTL responses [114].

## 5. Future Perspectives

Proof of concept studies are required to provide mechanistic understanding of the application of an effective type of adjuvant. This can be achieved by studying vaccine formulations composed of different adjuvants. For humoral immune responses, the use of the adjuvant should result in enhanced antibody formation (concentration or titre of vaccine-specific antibodies), antibody affinity (or avidity) and the functional consequences (opsonisation and phagocytosis, complement activation, neutralizing capacity, *etc.*). For cellular immune responses, on the other hand, adjuvants should modulate DC subsets and their cytokines, which drive CD4+ Th and CD8+ Tc subset differentiation and the associated cytokine production profiles of these effector cells. Animal models or *in vitro* analysis of human immune cells should enable the analysis of the protective and therapeutic capacity of vaccines based on different adjuvants. This capacity should be reflected in the efficacy of the induced vaccine-specific immune response, which includes bactericidal and neutralizing activity, opsonisation and phagocytosis capacity, antigen-presentation capacity, T-cell differentiation capacity and the quantity and quality of the resulting antibody production. Collectively, these studies will increase the understanding of the mode of action of the relevant adjuvants, but also monitoring of their safety profiles and their side effects *in vitro* and *in vivo*. Future studies will certainly unravel these events for distinct types of adjuvants in a systematic approach and will contribute to rational vaccine design.

## 6. Conclusions

Recent progress in the understanding of inflammation and innate immune responses (and their connection to the generation of adaptive immune defence) has resulted in a better understanding of the underlying principles for the mechanism of action of selective adjuvants. The simultaneous and integrated exposure to vaccine antigens and innate response modifiers, like PRR ligands (TLR, NOD, NLR), resulting in improved immune responses, provides the means to rationally design new vaccines with improved performance. This is urgently needed, in particular for cellular immunity-promoting vaccines and for mucosal vaccines.
